# Prostate Radiotherapy for Metastatic Hormone-sensitive Prostate Cancer: A STOPCAP Systematic Review and Meta-analysis

**DOI:** 10.1016/j.eururo.2019.02.003

**Published:** 2019-07

**Authors:** Sarah Burdett, Liselotte M. Boevé, Fiona C. Ingleby, David J. Fisher, Larysa H. Rydzewska, Claire L. Vale, George van Andel, Noel W. Clarke, Maarten C. Hulshof, Nicholas D. James, Christopher C. Parker, Mahesh K. Parmar, Christopher J. Sweeney, Matthew R. Sydes, Bertrand Tombal, Paul C. Verhagen, Jayne F. Tierney, the STOPCAP M1 Radiotherapy Collaborators

**Affiliations:** a Meta-analysis Group, MRC Clinical Trials Unit at UCL, London, UK; b Department of Urology, OLVG, Amsterdam, The Netherlands; c Department of Urology, Amsterdam UMC (VU), Amsterdam, The Netherlands; d MRC Clinical Trials Unit at UCL, London, UK; e The Christie and Salford Royal Hospitals, Manchester, UK; f Department of Radiotherapy, Amsterdam UMC (AMC), Amsterdam, The Netherlands; g Institute of Cancer and Genomic Sciences, University of Birmingham, Edgbaston, Birmingham, UK; h Royal Marsden Hospital, Sutton, Institute of Cancer Research, Sutton, UK; i Dana-Faber Cancer Institute, Boston, MA, USA; j Department of Urology, Cliniques Universitaires Saint Luc, Brussels, Belgium; k Department of Urology, Erasmus Medical Centre, Rotterdam, The Netherlands

**Keywords:** Prostate cancer, Radiotherapy, Metastases, Systematic review, Meta-analysis, Androgen deprivation therapy, Standard of care

## Abstract

**Background:**

Many trials are evaluating therapies for men with metastatic hormone-sensitive prostate cancer (mHSPC).

**Objective:**

To systematically review trials of prostate radiotherapy.

**Design, setting, and participants:**

Using a prospective framework (framework for adaptive meta-analysis [FAME]), we prespecified methods before any trial results were known. We searched extensively for eligible trials and asked investigators when results would be available. We could then anticipate that a definitive meta-analysis of the effects of prostate radiotherapy was possible. We obtained prepublication, unpublished, and harmonised results from investigators.

**Intervention:**

We included trials that randomised men to prostate radiotherapy and androgen deprivation therapy (ADT) or ADT only.

**Outcome measurements and statistical analysis:**

Hazard ratios (HRs) for the effects of prostate radiotherapy on survival, progression-free survival (PFS), failure-free survival (FFS), biochemical progression, and subgroup interactions were combined using fixed-effect meta-analysis.

**Results and limitations:**

We identified one ongoing (PEACE-1) and two completed (HORRAD and STAMPEDE) eligible trials. Pooled results of the latter (2126 men; 90% of those eligible) showed no overall improvement in survival (HR = 0.92, 95% confidence interval [CI] 0.81–1.04, *p* = 0.195) or PFS (HR = 0.94, 95% CI 0.84–1.05, *p* = 0.238) with prostate radiotherapy. There was an overall improvement in biochemical progression (HR = 0.74, 95% CI 0.67–0.82, *p* = 0.94 × 10^−8^) and FFS (HR = 0.76, 95% CI 0.69–0.84, *p* = 0.64 × 10^−7^), equivalent to ∼10% benefit at 3 yr. The effect of prostate radiotherapy varied by metastatic burden—a pattern consistent across trials and outcome measures, including survival (<5, ≥5; interaction HR = 1.47, 95% CI 1.11–1.94, *p* = 0.007). There was 7% improvement in 3-yr survival in men with fewer than five bone metastases.

**Conclusions:**

Prostate radiotherapy should be considered for men with mHSPC with a low metastatic burden.

**Patient summary:**

Prostate cancer that has spread to other parts of the body (metastases) is usually treated with hormone therapy. In men with fewer than five bone metastases, addition of prostate radiotherapy helped them live longer and should be considered.

## Take Home Message

This prospectively designed systematic review showed that prostate radiotherapy did not improve survival in unselected men. A difference in effect by metastatic burden was evident across included trials and outcomes; 3-yr survival increased by 7% in men with fewer than five bone metastases.

## 1 Introduction

Randomised controlled trials have evaluated, or are currently evaluating, promising therapies for metastatic hormone-sensitive prostate cancer (mHSPC), including prostate radiotherapy [1], [2]. Systematic reviews of these trial results can help determine effective treatments, but are usually planned after most trials have reported and focus on published results. Consequently, their design and conduct can be influenced by existing results, and they may not include enough data to produce reliable findings.

A new framework for adaptive meta-analysis (FAME) [5] defines review methods prospectively, prior to trial results being published. It also helps anticipate emerging trial results and identify the earliest opportunity for reliable meta-analysis [6], [7]. Results of the key trials investigating prostate radiotherapy were due, which could provide sufficient evidence about its effects.

## 2 Patients and methods

We aimed to assess the effects of adding prostate radiotherapy to androgen deprivation therapy (ADT) in men with mHSPC. We prespecified methods in a protocol prior to the results of eligible trials being known (PROSPERO registration: CRD42018096108).

### 2.1 Treatment comparisons

Some eligible trials are assessing the effects of prostate radiotherapy in conjunction with other agents. To allow for the possibility of an interaction between these different treatments, we wanted to review the effects of prostate radiotherapy, via two comparisons.

*Comparison A:* Prostate radiotherapy + ADT versus ADT
*Comparison B:* Prostate radiotherapy + other agent(s) + ADT versus (same) other agents(s) + ADT

### 2.2 Framework for adaptive meta-analysis

We applied key FAME principles to: (1) start the systematic review process whilst trials are ongoing or yet to report, (2) search comprehensively for all eligible trials, (3) liaise with trial teams to develop a detailed picture of these trials, (4) predict when sufficient results will be available for reliable meta-analysis, (5) conduct meta-analysis and interpret results taking account of any unavailable data, and (6) assess the value of updating.

### 2.3 Trial eligibility

Randomised controlled trials were eligible if they randomised men with mHSPC, starting or responding to first-line hormone therapy, and compared prostate radiotherapy plus ADT versus ADT. Trials including additional agents (eg, docetaxel, abiraterone) were also eligible, provided that the same additional agents were used in both treatment and control arms. Trials were ineligible if they included men who had stopped responding to first-line hormone therapy, those with castrate-refractory prostate cancer, or those in whom radiotherapy was administered to metastases.

### 2.4 Trial identification

We regularly searched systematically for all published, unpublished, and ongoing trials in mHSPC. With no language restrictions, we searched MEDLINE, EMBASE, clinicaltrials.gov, and Cochrane CENTRAL up to June 2018 (see Supplementary material). We also searched relevant conference proceedings (Supplementary Table 1) and reference lists of review articles, and identified trial reports/protocols.

Two reviewers independently assessed all unique records (L.H.R. and S.B.), obtained full papers or protocols for any trials deemed potentially eligible, and agreed on the final set of trials. We asked trialists to supplement this list, and provide updated status and reporting plans for their trials.

### 2.5 Outcome measures

The primary outcome was survival, defined as the time from randomisation to death from any cause. Secondary outcomes were progression-free survival (PFS), defined as the time from randomisation to first symptomatic clinical or radiological progression or death (excluding biochemical progression); biochemical progression, defined as the time from randomisation to first biochemical (prostate-specific antigen [PSA]) progression; and failure-free survival (FFS), defined as the time from randomisation to first biochemical, clinical, or radiological progression. We also aimed to describe acute toxicity on the radiotherapy arm.

### 2.6 Data collection

We sought information from investigators on trial accrual period, number of patients, age, PSA, performance status, T and N category, location and number of metastases, disease history, Gleason sum score, type of hormone therapy, volume of disease, prostate radiotherapy dose, and toxicity. We also sought overall results for survival, PFS, FFS, and biochemical progression according to our prespecified definitions, as well as results for survival, PFS, and FFS by patient subgroups (age, performance status, clinical T stage, nodal status, Gleason sum score, type of hormone therapy, disease history, location of metastases, number of bone metastases, and volume of disease by the CHAARTED [8] and LATITUDE [9] definitions).

We assessed the risk of bias [10] of included trials based on sequence generation, allocation concealment, completeness of outcome data, and selective outcome reporting, using information obtained from trial protocols, manuscripts, or investigators.

### 2.7 Analysis

#### 2.7.1 Planning reliable meta-analyses

In early 2018, we identified three trials eligible for comparison A: STAMPEDE [11], HORRAD [12], and PEACE-1 (NCT01957436; Table 1). We found that the ongoing PEACE-1 trial was not due to report for some years, but the STAMPEDE and HORRAD trials would report in late 2018. We anticipated that each would have a median follow-up of at least 3 yr and would provide results for 2140 men; 90% of those eligible. Based on typical 3-yr survival in mHSPC [9], [13], we predicted that these would give approximately 66% and 99% power to detect 5% and 10% absolute differences in 3-yr survival, respectively. Thus, we planned an early, potentially definitive meta-analysis.

**Table 1:** Characteristics of trials (or parts of trials) eligible for comparison A

Trial	Years of accrual	Number of men randomised	De novo or relapsed M1?	Treatment	Control	Median follow-up (survival)
				Radiotherapy	ADT	
**Radiotherapy** **+** **ADT vs ADT**						
STAMPEDE A1 [11] (arm H vs arm A)	2013–2016	1694	De novo	36 Gy, 6 fractions over 6 wk or 55 Gy, 20 fractions over 4 wk	ADT (LHRH agonist or antagonist or orchiectomy)	41.9 mo
HORRAD [12]	2004–2014	432	De novo	70 Gy, 35 fractions over 7 wk or 57.76 Gy, 19 fractions over 6 wk	ADT (LHRH agonist or orchiectomy)	47 mo
PEACE-1A1 (NCT01957436)	2013–2018*	234	De novo	74 Gy, 37 fractions within 7–8 wk	ADT (LHRH agonist or antagonist or orchiectomy)	Not yet available

ADT = androgen deprivation therapy; LHRH = luteinising hormone-releasing hormone. *PEACE-1 closed to accrual between submission and acceptance of the manuscript

Two trials were eligible for comparison B: STAMPEDE [11] and PEACE-1 (NCT01957436), potentially including 1299 men (Table 2). However, as only the STAMPEDE results for 367 men randomised to receive docetaxel as part of the standard of care were anticipated in 2018, a definitive meta-analysis of comparison B is planned later.

**Table 2:** Characteristics of trials (or parts of trials) eligible for comparison B

**Trial**	**Years of accrual**	**De novo or relapsed M1?**	**Treatment**	**Control**			**Number of patients accrued**
			**Radiotherapy**	**Docetaxel**	**Abiraterone/** **Prednisone**	**ADT**	
**B1 Radiotherapy + abiraterone + ADT versus abiraterone + ADT**							
PEACE-1B1 (NCT01957436)	2013-2018*	De novo	74Gy, 37 fractions within 7 to 8 wk	-	Abiraterone 1000mg/day Prednisone 10mg/day	ADT LHRH agonist or antagonist or orchiectomy	229
**B2 Radiotherapy + docetaxel + ADT versus docetaxel + ADT**							
STAMPEDE B2[11] (Arm H vs Arm A)	2015-2016	De novo	36Gy, 6 fractions over 6 weeks or 55Gy, 20 fractions over 4 wk	According to local protocol or 75mg/m2 every 3 wk for 6 cycles	-	ADT LHRH agonist or antagonist or orchiectomy	367
PEACE-1B2 (NCT01957436)	2013-2018*	De novo	74Gy, 37 fractions within 7 to 8 wk	75mg/m2 every 3 wk for 6 cycles	-	ADT LHRH agonist or antagonist or orchiectomy	355
**B3 Radiotherapy + abiraterone + docetaxel + ADT versus abiraterone + docetaxel + ADT**							
PEACE-1B3 (NCT01957436)	2013-2018*	De novo	74Gy, 37 fractions within 7 to 8 wk	75mg/m2 every 3 wk for 6 cycles	Abiraterone 1000mg/day Prednisone 10mg/day	ADT LHRH agonist or antagonist or orchiectomy	355

ADT = androgen deprivation therapy; LHRH = luteinising hormone-releasing hormone. *PEACE-1 closed to accrual between submission and acceptance of the manuscript

#### 2.7.2 Measuring treatment effects

For time-to-event outcome measures (overall survival, PFS, FFS, and biochemical progression) and hazard ratios (HRs) were combined using the fixed-effect model[14]. Chi-square tests and *I*^2^ statistic were used to assess statistical heterogeneity [15]. We aimed to summarise grade 1–5 acute bladder and bowel toxicities in the radiotherapy arm.

We also planned analyses of the effects of prostate radiotherapy on overall survival by prespecified subgroups defined by age (<70, >70 yr), performance status (0, 1+), nodal status (N0, N+), Gleason sum score (<8, ≥8), type of ADT (orchiectomy, luteinising hormone-releasing hormone [LHRH] agonist, LHRH antagonist), disease history (de novo metastatic disease, relapsed after prior local therapy with curative intent), location of metastases (bone, visceral, other), number of bone metastases (0, 1–3, 4–9, >9), and volume of disease (high volume, low volume). If there were insufficient numbers of men within subgroups, we collapsed them to achieve groups of a reasonable size, or did not perform subgroup analyses. Where categories were incompatible across trials or were not those predefined for the meta-analysis, we requested additional results from the investigators.

For each subgroup, we calculated interaction HRs separately for each trial. For subgroups of two categories, interaction HRs were calculated as the ratio of the two subgroup HRs. For subgroups of three ordered categories, interaction HRs were estimated using a weighted linear regression of subgroup HRs, with error variances assumed to be known. The interaction HRs were combined across trials using a fixed-effect meta-analysis [16], [17]. If there was any evidence of an interaction, we replicated the relevant subgroup analysis on PFS and FFS, in order to support or refute the findings. We subsequently estimated “pooled” HRs by subgroup, consistent with the pooled interaction HR, using multivariate meta-analysis with the variance estimated using the delta method. Absolute differences in outcome at 3 yr were derived from these subgroup HRs and a representative control-group event rate. All *p* values are two sided. Analyses were carried out using [3] Stata version 15.1.[4]

#### 2.7.3 Network meta-analysis of current therapies

Previously, we compared the relative effects of recent therapies combined with ADT in a network meta-analysis [18]. If the survival results of comparison A were deemed sufficiently reliable, we would include them in an updated network meta-analysis.

## 3 Results

### 3.1 Characteristics of eligible trials

Our searches retrieved 19,830 unique records, and seven mHSPC trials that were potentially eligible for comparison A. Four trials were excluded: two because radiotherapy was administered to metastases as well as the prostate ([19] and NCT02913859), one because men did not receive ADT (NCT02680587), and one because surgery or radiotherapy was allowed as local treatment (NCT01751438), leaving three eligible trials (Supplementary Fig. 1).

As the ongoing trial only closed to recruitment at the end of 2018, the meta-analysis includes results of the two completed and reported trials (HORRAD [12] and STAMPEDE [11]). These include fewer men than anticipated (2126/2360), because the number of individuals recruited to HORRAD was fewer than planned (Table 1). but still represent about 90% of men eligible for comparison A. (Table 1).

HORRAD randomised 432 men between 2004 and 2014, and STAMPEDE 1694 men between 2013 and 2016, to prostate radiotherapy and ADT versus ADT (Table 3). Median follow-up was 47 mo in HORRAD (interquartile range [IQR]: 36–68 mo) and 41.9 mo in STAMPEDE (IQR: 31–49 mo).

**Table 3:** Characteristics of patients at randomisation

	HORRAD [11]		STAMPEDE [12]^a^	
	ADT	RT + ADT	ADT	RT + ADT
Number of patients	216	216	845	849
Disease history				
Newly diagnosed M1	216 (100)	216 (100)	845 (100)	849 (100)
Type of ADT, *n* (%)				
Orchiectomy	4 (2)	1 (<1)	0	0
LHRH agonist	212 (98)	210 (97)	672 (80)	684 (81)
LHRH antagonist	0	0	165 (19)	159 (18)
Missing	0	5 (2)	8 (1)	6 (<1)
Time from initial diagnosis (mo), *n* (%)				
Median (IQR)	<1 (2–5 wk)	<1 (2–6 wk)	2.4 (1.8–3.1)	2.4 (1.8, 3.1)
Range	0–42	0–25	0–114.8	0–41.9
Missing	0	0	5	14
Time to ADT start (wk)				
Median (IQR)	−1 (−3, 0)	−1 (−3, 0)	−1.7 (−2.3, −1.1)	−1.7 (−2.3, −1.1)
Range	−17 to 2	−13 to 2	−2.8 to 1.1	−2.8 to 0.3
Missing	2	4	0	1
Age (yr)				
Median (IQR)	67 (61–71)	67 (62–71)	68 (63, 73)	68 (63, 73)
Range	47–85	47–79	37–84	45–87
WHO/ECOG performance status				
0	176 (82)	187 (87)	597 (71)	603 (71)
1+	40 (18)	29 (13)	248 (29)	246 (29)
PSA (ng/ml), *n* (%)				
Median (IQR)	149 (50–483)	125 (48–433)	100 (30, 311)	96 (33,299)
Range	4–6991	8–14,000	1–20,590	1–11,156
T category				
T0	0	0	0	2 (<1)
T1	5 (2)	7 (3)	11 (1)	11 (1)
T2	20 (10)	33 (15)	69 (8)	73 (9)
T3	128 (59)	125 (58)	474 (56)	500 (59)
T4	59 (27)	51 (24)	222 (26)	198 (23)
T*x*	4 (2)	0	69 (9)	65 (7)
N category, *n* (%)				
N0	–	–	293 (35)	292 (34)
N+	–	–	500 (59)	498 (59)
Nx	216 (100)	216 (100)	52 (6)	59 (7)
Gleason sum score, *n* (%)				
<8	71 (33)	73 (34)	151 (18)	144 (17)
≥8	144 (66)	142 (65)	668 (79)	665 (78)
Unknown	1 (<1)	1 (<1)	26 (3)	40 (5)
Number of bone metastases, *n* (%)				
<5	71 (33)	89 (41)	404 (48)	399 (47)
>5	145 (67)	127 (58)	397 (47)	393 (46)
Unknown	–	–	44 (5)	57 (7)
Metastatic burden (HORRAD definition^b^), *n* (%)				
Low burden	35 (16)	39 (18)	385 (46)	387 (46)
High burden	181 (84)	177 (82)	416 (49)	405 (48)
Unknown	–	–	44 (5)	57 (6)
Planned RT dose, *n* (%)				
36 Gy in 6 fr over 6 wk, *n* (%)	NA	NA	NA	416 (49)
55 Gy in 20 fr over 4 wk *n* (%)	NA	NA	NA	433 (51)
70 Gy in 35 fr over 7 wk, *n* (%)	NA	176 (82)	NA	NA
57.76 Gy in 19 fr over 4 wk, *n* (%)	NA	26 (12)	NA	NA
Unknown, *n* (%)		14 (6)		

ADT = androgen deprivation therapy; fr = fraction; IQR = interquartile range; LHRH = luteinising hormone-releasing hormone; NA = not available; RT = radiotherapy.

a Based on the participants who did not receive docetaxel as part of standard of care.

b Low = Gleason sum score <9 and <5 bone lesions and PSA <142 (HORRAD median).

All men were classified as having newly diagnosed mHSPC and were receiving long-term ADT for the first time, mostly LHRH-based therapy (>99%). Across the two trials, men were aged similarly (HORRAD, median age 67 yr; STAMPEDE, median age 68 yr); most had World Health Organisation/ECOG performance status 0 (HORRAD, 84%; STAMPEDE, 71%) and a Gleason sum score of ≥8 (HORRAD, 66%; STAMPEDE, 79%). All men recruited to the HORRAD trial had bone metastases, while 89% of men in the STAMPEDE trial had bone metastases with (5%) or without (84%) visceral metastases. As the HORRAD trial did not collect data on nonbone metastases, it was not possible to use the CHAARTED [8] or LATITUDE [9] trial definitions of disease volume. However, the STAMPEDE team were able to reclassify patients according to the HORRAD definition [20] (low volume: Gleason sum score <9, fewer than five bone lesions, and PSA ≤142; the HORRAD median). Few men in the HORRAD trial (17%) had low-volume disease compared with around half of the men in the STAMPEDE trial (45%).

In HORRAD, planned prostate radiotherapy was initially 70 Gy in 35 fractions over 7 wk (82%), with an alternative schedule of 57.76 Gy in 19 fractions over 6 wk (12%) added subsequently, which was considered biologically equivalent to 70 Gy in 7 wk (6% unknown). In STAMPEDE, clinicians had the choice of radiotherapy dose: 36 Gy in six fractions over 6 wk (49%) or 55 Gy in 20 fractions over 4 wk (51%).

Based on randomisation sequence generation, allocation concealment, completeness of outcome data, and selective outcome reporting, both trials were judged to be at a low risk of bias (Supplementary Table 2).

### 3.2 Overall treatment effects

**Fig. 1:**
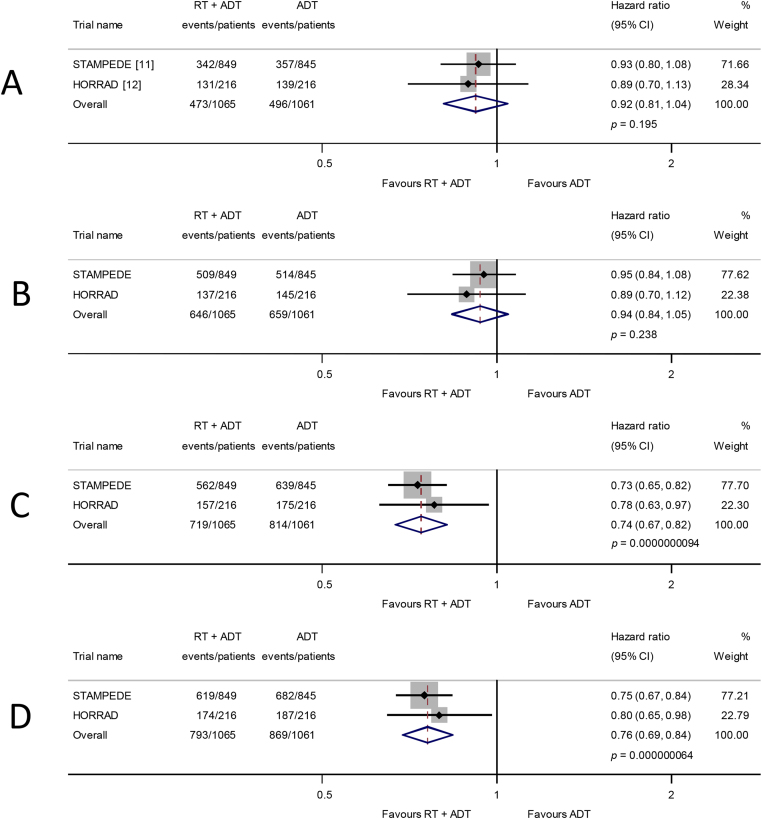
Effect of adding prostate radiotherapy to ADT on (A) survival, (B) progression-free survival, (C) biochemical progression, and (D) failure-free survival in men with mHSPC. Each filled square denotes the HR for that trial comparison, with the horizontal lines showing the 95% confidence interval (CI). The size of the square is directly proportional to the amount of information contributed by a trial. The diamond represents a (fixed-effect) meta-analysis of the trial HRs, with the centre of this diamond indicating the HR and the extremities the 95% CI. ADT = androgen deprivation therapy; HR = hazard ratio; RT = radiotherapy.

Survival results are based on all 2126 men (969 deaths) from HORRAD and STAMPEDE. Overall, there was no evidence that the addition of prostate radiotherapy to ADT improved survival (HR = 0.92, 95% confidence interval [CI] 0.81–1.04, *p* = 0.195; heterogeneity chi-square = 0.08, degree of freedom = 1, *p* = 0.78; Fig. 1).

The PFS results based on all men (1305 events) also provided no clear evidence that, overall, prostate radiotherapy extended PFS (HR = 0.94, 95% CI 0.84–1.05, *p* = 0.238; Fig. 1). Although, in the HORRAD trial, biochemical progression was defined as the time between diagnosis and a PSA increase after the initiation of ADT of >50% of the lowest PSA value after the start of treatment (with a minimum of 1 ng/ml), and in the STAMPEDE trial as a rise above the lowest PSA within 24 wk of enrolment of 50% to at least 4 ng/ml, we considered them sufficiently compatible to combine. Based on all men and 1533 events, we observed a highly statistically significant benefit of prostate radiotherapy (HR = 0.74, 95% CI 0.67–0.82, *p* = 0.94 × 10^−8^; Fig. 1) in biochemical progression, which translates to an absolute improvement of 11 (7–14)% at 3 yr from 25% to 36%. The FFS results based on all men (1662 events) were very similar (HR = 0.76, 95% CI 0.69–0.84, *p* = 0.64 × 10^−7^; Fig. 1).

**Fig. 2:**
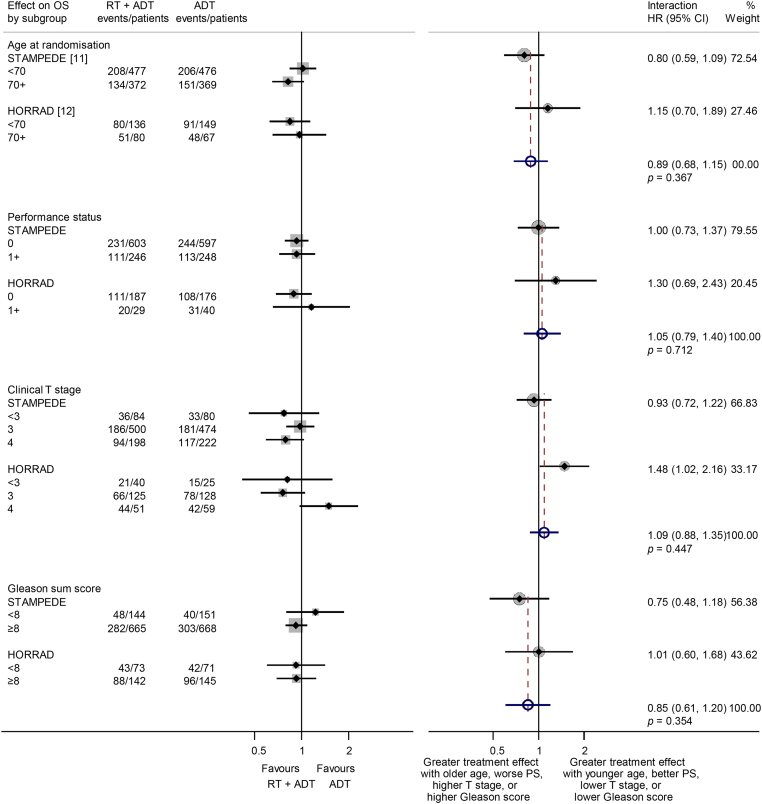
Effect of adding prostate radiotherapy to ADT on survival by patient age at randomisation, performance status, clinical T stage, and Gleason sum score. Each filled square denotes the HR for each subgroup of men defined by, age at randomisation, performance status, clinical T stage, and Gleason sum score within each trial, with the horizontal lines showing the 95% confidence interval (CI). The size of the square is directly proportional to the amount of information contributed by a subgroup. Each filled circle denotes the HR for the interaction between the effect of radiotherapy and these subgroups for each trial, with the horizontal lines showing the 95% CI. The size of each circle is directly proportional to the amount of information contributed by a trial. The open circle represents a (fixed-effect) meta-analysis of the interaction HRs, with the horizontal line showing the 95% CI. ADT = androgen deprivation therapy; HR = hazard ratio; RT = radiotherapy.

Toxicity results are not yet available for HORRAD. Based on the results collected from STAMPEDE, 4% of men who received prostate radiotherapy had severe acute bladder toxicity, and 1% had severe acute bowel toxicity (RTOG scale). Reported STAMPEDE results showed that 4% of men had severe late effects.

Based on shorter median follow-up (21.3 mo) and only 367 men, the STAMPEDE survival results for men planned for docetaxel (HR = 0.81, 95% CI 0.49–1.34, *p* = 0.379) were broadly similar to the results of comparison A.

### 3.3 Treatment effects by patient characteristics

As all men were newly diagnosed and most received LHRH-based ADT; planned survival analyses by disease history and type of ADT were not possible. There was no evidence that the effect of prostate radiotherapy on survival varied by our prespecified subgroups: age (interaction HR = 0.89, 95% CI 0.68–1.15, *p* = 0.367), performance status (interaction HR = 1.05, 95% CI 0.79–1.40, *p* = 0.712), clinical T-stage (interaction HR = 1.09, 95% CI 0.88–1.35, *p* = 0.447), and Gleason sum score (interaction HR = 0.85, 95% CI 0.61–1.20, *p* = 0.354; Fig. 2).

**Fig. 3:**
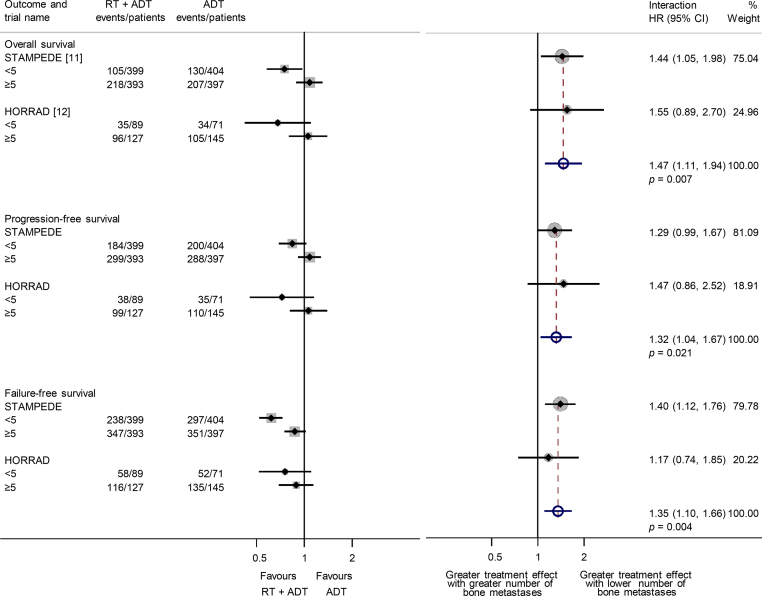
Effect of adding prostate radiotherapy to ADT on survival, progression-free survival, and failure-free survival (exploratory) by the number of bone metastases. ADT = androgen deprivation therapy; HR = hazard ratio; RT = radiotherapy.

As the HORRAD trial collected the number of bone metastases in three prespecified categories (<5, 5–15, and >15), and the STAMPEDE trial collected the absolute number of metastases up to 9 and then >9, we obtained compatible results from both trials for our planned analyses (<5, ≥5). The effect of prostate radiotherapy on survival varied by the number of bone metastases (interaction HR = 1.47, 95% CI 1.11–1.94, *p* = 0.007; Fig. 3), with a benefit seen in men with fewer than five bone metastases (HR = 0.73, 95% CI 0.58–0.92, *p* = 0.0071), which translates to an absolute improvement of 7% (95% CI 2–11%) at 3-yr survival (from 70% to 77%). There was no clear evidence of an effect among men with five or more bone metastases (HR = 1.07, 95% CI 0.92–1.26, *p* = 0.37). A similar planned analysis of PFS (interaction HR = 1.32, 95% CI 1.04–1.67, *p* = 0.021; Fig. 3) and an exploratory analysis of FFS (interaction HR = 1.35, 95% CI 1.10–1.66, *p* = 0.004; Fig. 3) gave comparable results.

An exploratory analysis using the HORRAD definition [20] provided evidence that the effect of prostate radiotherapy on survival varied by volume of disease (interaction HR = 1.44, 95% CI 1.07–1.95, *p* = 0.017; Supplementary Fig. 2), which was supported by similar analyses of PFS (HR = 1.28, 95% CI 1.00–1.64, *p* = 0.054; Supplementary Fig. 2) and FFS (HR = 1.40, 95% CI 1.12–1.74, *p* = 0.003; Supplementary Fig. 2).

#### 3.3.1 Network meta-analysis of all current therapies

As the effect of prostate radiotherapy on survival was influenced by metastatic burden, this would need to be accounted for in the planned update of the network meta-analysis of recent therapies for mHSPC [18], and such methods are still in development [21]. It would also require the collection and analysis of individual participant data (IPD) from all trials.

## 4 Discussion

### 4.1 Summary of results

Prostate radiotherapy did not clearly improve survival or PFS in unselected men with mHSPC. However, there was a clear difference in the effect by metastatic burden on survival, with an absolute improvement of 7% in 3-yr survival in men who had four or fewer bone metastases. There was no evidence that the effect of prostate radiotherapy on survival varied by other patient or disease characteristics. Prostate radiotherapy improved 3-yr biochemical progression and FFS by ∼10% in unselected men, but the size of effect varied by metastatic burden.

### 4.2 Strengths

Based on 90% of all men randomised to prostate radiotherapy plus ADT versus ADT, we have shown that the effect of prostate radiotherapy on survival varies by metastatic burden. Despite different recruitment periods, radiotherapy approaches, and proportions of men with low and high metastatic burdens, this pattern was remarkably and reassuringly consistent across trials and outcomes. As a prospectively designed FAME review, all methods were published (unless otherwise specified) before trial results were known. This includes the preplanned subgroup analyses by metastatic burden, albeit that we had to collapse subgroup categories. We were able to anticipate when the results of STAMPEDE and HORRAD were due, allowing us to align the review with publication of their results [11], [12]. By obtaining unpublished trial results, we could harmonise outcome and subgroup definitions and conduct additional analyses. Hence, we have been able to provide a more timely, reliable, and thorough synthesis of the effects of prostate radiotherapy than is usually possible with summary results [5].

### 4.3 Limitations

Only two of the relevant trials are included, but the 234 eligible men from the recently completed PEACE-1 trial (Table 1) represent just 10% of the total, and so its results are unlikely to materially affect our findings. While the STAMPEDE trial heavily influences the results, the HORRAD trial has longer follow-up and adds considerable weight to the analyses of all outcome measures (23–28%; Fig. 1), including the survival analysis by metastatic burden (25%; Fig. 3). Therefore, until internationally agreed, optimised definitions of the metastatic burden and the oligometastatic state are determined [22], the number of bone metastases alone could help identify groups of men who might benefit from prostate radiotherapy.

### 4.4 Context

Results from PEACE-1 in combination with the current results of STAMPEDE will provide the first substantive evidence of how prostate radiotherapy works in conjunction with docetaxel and/or abiraterone. A new trial (SWOG S1802, NCT03678025) of standard systemic therapy with or without definitive treatment (surgery or radiotherapy) may also contribute to this comparison, if it stratifies by definitive treatment. Three trials (TROMBONE [ISRCTN 15704862], g-RAMPP [NCT02454543], and SIMCAP [NCT03456843]) are investigating whether radical prostatectomy offers an alternative to radical radiotherapy in this setting, and two trials [19] (NCT02913859) and a new STAMPEDE arm are evaluating the effects of administering radiotherapy to metastatic sites as well as the prostate.

### 4.5 Implications

The collection of IPD from relevant trials could help determine which men with mHSPC benefit more or less from prostate radiotherapy and what the optimal definition of metastatic burden might be. A comprehensive repository of IPD from all modern mHSPC trials (STOPCAP M1 IPD repository) is being established with funding from MRC and Prostate Cancer UK to tackle these and other important clinical uncertainties (http://www.stopcapm1.org/). However, applying the review findings in settings where newer imaging techniques (eg, prostate-specific membrane antigen positron emission tomography) are available could be problematic, as men currently classed as having a low metastatic burden may be reclassified as having a greater number of metastases. Questions also remain regarding the timing and optimal dose of radiotherapy.

## 5 Conclusions

The addition of prostate radiotherapy to ADT should be considered for men with mHSPC who have four or fewer bone metastases.

***Author contributions***: Sarah Burdett had full access to all the data in the study and takes responsibility for the integrity of the data and the accuracy of the data analysis.  

*Study concept and design:* Burdett, Tierney, Fisher, Rydzewska, Vale.

*Acquisition of data:* Burdett.

*Analysis and interpretation of data:* Burdett, Fisher, Tierney.

*Drafting of the manuscript:* Burdett, Tierney.

*Critical revision of the manuscript for important intellectual content:* Burdett, Tierney, Fisher, Rydzewska, Vale, Tombal, Parmar, Sweeney, van Andel, Boevé, Verhagen, Hulshof, Clarke, James, Ingleby, Parker, Sydes.

*Statistical analysis:* Fisher.

*Obtaining funding:* None.

*Administrative, technical, or material support:* None.

*Supervision:* None.

*Other:* (Provision of trial results) Boevé, Ingleby.  

***Financial disclosures:*** Sarah Burdett certifies that all conflicts of interest, including specific financial interests and relationships and affiliations relevant to the subject matter or materials discussed in the manuscript (eg, employment/affiliation, grants or funding, consultancies, honoraria, stock ownership or options, expert testimony, royalties, or patents filed, received, or pending), are the following: No conflicts of interest for Sarah Burdett, Liselotte M. Boevé, Jayne F. Tierney, David J. Fisher, Larysa H. Rydzewska, Claire L. Vale, Fiona C. Ingleby, George van Andel, Maarten C. Hulshorf and Paul C. Verhagen. Noel W. Clarke reports personal fees from Janssen outside the submitted work. Nicholas D. James reports advisory board fees from Sanofi and Novartis, outside the submitted work; and grants, personal fees, non-financial support, advisory board fees, speaker fees and travel fees from Janssen, outside the submitted work. Christopher C. Parker reports a research grant, personal fees, and advisory board fees from Bayer, outside the submitted work; advisory board fees from AAA, outside the submitted work and personal fees and speaker fees from Janssen, outside the submitted work. Mahesh K. Parmar reports educational grants for the STAMPEDE trial from Astellas, Clovis Oncology, Novartis, Pfizer, and Sanofi, ouside the submitted work. The Unit he directs also receives educational grants and other non-financial support from a large number of different companies. Christopher J. Sweeney reports ownership Leuchemix; advisory board participation for Astellas/Medivation, Pfizer, Astra Zeneca, Sanofi, Janssen and Bayer; Grants from Janssen, Astellas/Medivation, Sanofi, Janssen, Sotio; and consultancy for Astellas/Medivation, Pfizer, Sanofi, Janssen, BIND, Bayer, Genentech and Amgen outside the submitted work. Matt R. Sydes reports grants and non-financial support from Astellas, Clovis Oncology, Novartis, Pfizer and Sanofi, outside the submitted work; personal fees from Eli Lilly, outside the submitted work; and grants, personal fees and non-financial support from Janssen, outside the submitted work. Bertrand Tombal reports advisory board fees from Amgen, Astellas, Ferring, Sanofi, Bayer and Myovant, outside the submitted work.  

***Funding/Support and role of the sponsor*****:** The STOPCAP Project Management Group was funded by the UK Medical Research Council (MC_UU_12023/25) and Prostate Cancer UK (RIA16-ST2-020). HORRAD was funded by an educational grant from Ipsen and AstraZeneca. STAMPEDE was funded by Cancer Research UK (CRUK_A12459), Medical Research Council (MRC_MC_UU_12023/25), Swiss Group for Cancer Clinical Research, Astellas, Clovis Oncology, Janssen, Novartis, Pfizer, and Sanofi-Aventis. The funders (Prostate Cancer UK, RIA16-ST2-020; UK Medical Research Council, MC_UU_12023/25) had no role in the study design, data collection, data analysis, data interpretation, or writing of the report.  

***Acknowledgements:*** The STOPCAP M1 Radiotherapy Collaborators thank all patients who participated in the trials and contributed to this research. The meta-analysis would not have been possible without their participation or without the institutions and collaborative groups that carried out the trials, including a great many trial sites. We thank the HORRAD and STAMPEDE trial teams for supplying prepublication results, and additional analysis to facilitate meta-analysis, including Christopher Brawley for second programming of key STAMPEDE analyses. We also thank Karim Fizazi for information regarding PEACE-1 trial accrual.
