# Supplementary material for: Prostate Radiotherapy for Metastatic Hormone-sensitive Prostate Cancer: A STOPCAP Systematic Review and Meta-analysis
Source: Eur Urol. 2019 Jul;76(1):115–24. doi: 10.1016/j.eururo.2019.02.003 (PMC6575150; doi:10.1016/j.eururo.2019.02.003)
Supplement: Supplementary file 1 [file mmc1.docx]

**Supplementary material**

**Search Strategies**

**MEDLINE Search strategy**

Cochrane Highly Sensitive Search Strategy for identifying RCTs in MEDLINE: sensitivity and precision maximising version (2008 revision)^1^; Ovid format

**RCT filter MEDLINE**

1. randomi*ed controlled trial.pt.
2. controlled clinical trial.pt.
3. randomi*ed.ab.
4. placebo.ab.
5. clinical trials as topic.sh.
6. randomly.ab.
7. trial.ti.
8. 1 or 2 or 3 or 4 or 5 or 6 or 7
9. exp animals/ not humans.sh.
10. 8 not 9 (878394)

**AND - Terms specific to prostate cancer:**

1. exp Prostatic Neoplasms/
2. (prostat$ adj3 adeno$).mp.
3. (prostat$ adj3 malignan$).mp.
4. (prostat$ adj3 canc$).mp.
5. (prostat$ adj3 carcinoma$).mp.
6. (prostat$ adj3 tumo?r$).mp.
7. (prostat$ adj3 neoplas$).mp.
8. 11 or 12 or 13 or 14 or 15 or 16 or 17

**AND - Terms specific to metastatic disease**

1. exp Neoplasm Metastasis/
2. metastatic.mp.
3. exp Bone Neoplasms/
4. (osseous metastasis or osseous metastases).mp.
5. ((bone$ or skelet$ or osseous or osteo$) adj3 metast$).mp.
6. (metast$ adj3 prostat$).mp.
7. (advanced$ adj3 prostat$).mp.
8. 19 or 20 or 21 or 22 or 23 or 24 or 25

**AND - Combine terms for prostate cancer, metastatic disease, and RCTs**

1. 10 and 18 and 26

***/*** *means all subheadings were selected;* ***ab****=abstract;* ***mp****=free text search for a term;* ***pt*** *= publication type;* ***sh****=subject heading;* ***ti****=title*

**EMBASE Search strategy**

Best Optimisation of Sensitivity and Specificity Search^2^; Ovid format

**RCT filter EMBASE**

1. randomi*.tw.
2. placebo.mp.
3. double-blind.tw.
4. 1 or 2 or 3

**AND - Terms specific to prostate cancer:**

1. exp prostate carcinoma/
2. exp prostate cancer/
3. exp prostate tumor/
4. (prostat* adj3 adeno*).ti,ab,kw.
5. (prostat* adj3 malignan*).ti,ab,kw.
6. (prostat* adj3 canc*).ti,ab,kw.
7. (prostat* adj3 carcinoma*).ti,ab,kw.
8. (prostat* adj3 tumo?r*).ti,ab,kw.
9. (prostat* adj3 neoplas*).ti,ab,kw.
10. 5 or 6 or 7 or 8 or 9 or 10 or 11 or 12 or 13

**AND - Terms specific to metastatic disease:**

1. bone metastasis/
2. bone cancer/
3. metastasis/
4. metastat*.ti,ab,kw.
5. (osseous metastasis or osseous metastases).ti,ab,kw.
6. ((bone* or skelet* or osseous or osteo*) adj3 metast*).ti,ab,kw.
7. (metast* adj3 prostat*).ti,ab,kw.
8. (advanced* adj3 prostat*).ti,ab,kw.
9. 15 or 16 or 17 or 18 or 19 or 20 or 21 or 22

**AND Combine terms for RCTs, prostate cancer and metastatic disease**

1. 4 and 14 and 23

***/*** *means all subheadings were selected;* ***ab*** *=abstract;* ***ti*** *=title;* ***kw****=keyword;* ***mp****=free text search for a term;* ***tw****=text word*

**CENTRAL**

[RCT filter not required for CENTRAL (all RCTs)]

**Terms specific to prostate cancer:**

1. MeSH descriptor: [Prostatic Neoplasms] explode all trees
2. (prostat* near/3 adeno*)
3. (prostat* near/3 malignan*)
4. (prostat* near/3 canc*)
5. (prostat* near/3 carcinoma*)
6. (prostat* near/3 tumor*)
7. (prostat* near/3 neoplas*)
8. (prostat* near/3 metast*)
9. #1 or #2 or #3 or #4 or #5 or #6 or #7 or #8 or #9

**AND - Terms specific to metastatic disease:**

1. MeSH descriptor: [Neoplasm Metastasis] explode all trees
2. MeSH descriptor: [Bone Neoplasms] explode all trees
3. (metastatic or metastasis or metastases)
4. ((bone* or skelet* or osseous or osteo*) near/3 metast*)
5. (metast* near/3 prostat*)
6. (advanced* near/3 prostat*)
7. #10 or #11 or #12 or #13 or #14 or #15

**AND Combine terms for RCTs, prostate cancer and metastatic disease**

1. #9 and #16

**Clinicaltrials.gov**

Search terms: interventional studies [Study type] AND Phase II/III [Phase] AND prostate cancer [Condition] AND metastatic [Condition]

1. Lefebvre C, Manheimer E, Glanville J, on behalf of the Cochrane Information Retrieval Methods Group. Searching for studies. In: Higgins JPT, Green S, eds. Cochrane Handbook for Systematic Reviews of Interventions. Chichester: John Wiley & Sons Ltd; 2008: 95-150.

2. Wong SS, Wilczynski NL, Haynes RB. Developing optimal search strategies for detecting clinically sound treatment studies in EMBASE. *Journal of the Medical Library Association* 2006; **94**(1): 41-7.
