# Supplementary material for: Prostate Radiotherapy for Metastatic Hormone-sensitive Prostate Cancer: A STOPCAP Systematic Review and Meta-analysis
Source: Eur Urol. 2019 Jul;76(1):115–24. doi: 10.1016/j.eururo.2019.02.003 (PMC6575150; doi:10.1016/j.eururo.2019.02.003)
Supplement: Supplementary file 2 [file mmc2.docx]

**Supplementary Table 1: Sources searched for eligible trials**

| ***Sources searched*** | **Scope of search** |
| --- | --- |
| ***Electronic databases*** |  |
| MEDLINE | 1966-2018 |
| EMBASE | 1982-2018 |
| ***Trial registers*** |  |
| Cochrane Central Register of Controlled Trials (CENTRAL) | All records |
| ClinicalTrials.gov | All records |
| ***Conference proceedings (searched electronically)*** |  |
| American Society of Clinical Oncology (ASCO) | 2004-2018 |
| American Society of Clinical Oncology Genitourinary Meeting (ASCO GU) | 2009-2018 |
| European Society of Medical Oncology (ESMO) | 2004-2017 |
| European Cancer Conference Organization (ECCO) | 2004-2017 |
| American Urological Association (AUA) | 2008-2018 |
| European Association of Urology (EAU) | 2004-2018 |
| ***Conference proceedings (searched by hand)*** |  |
| American Society of Clinical Oncology (ASCO) | 1990-2003 |
| ***Additional handsearching*** |  |
| Trial report / review bibliographies |  |
| Direct contact with experts in the field |  |

**Supplementary Table 2: Assessment of risk of bias**[^10^](#_ENREF_15)

| **Trial ID** | **Adequate sequence generation** | **Allocation concealment** | **Incomplete outcome data addressed** | **Free of selective reporting** |
| --- | --- | --- | --- | --- |
| HORRAD[^12^](#_ENREF_17) | Patients assigned in a 1:1 ratio using a restricted block wise randomisation  **Low risk** | Randomisation done centrally by an independent trial office  **Low risk** | All randomised patients included in analyses  **Low risk** | All outcomes of interest reported  **Low risk** |
| STAMPEDE[^11^](#_ENREF_16) | A minimisation method with a random element of 80% was used to stratify for a number of clinically important factors  **Low risk** | Central telephone randomisation using a computer programme  **Low risk** | All randomised patients included in analyses  **Low risk** | All outcomes of interest reported  **Low risk** |
