# Supplementary material for: Prostate Radiotherapy for Metastatic Hormone-sensitive Prostate Cancer: A STOPCAP Systematic Review and Meta-analysis
Source: Eur Urol. 2019 Jul;76(1):115–24. doi: 10.1016/j.eururo.2019.02.003 (PMC6575150; doi:10.1016/j.eururo.2019.02.003)
Supplement: Supplementary file 3 [file mmc3.pptx]

## Slide 1
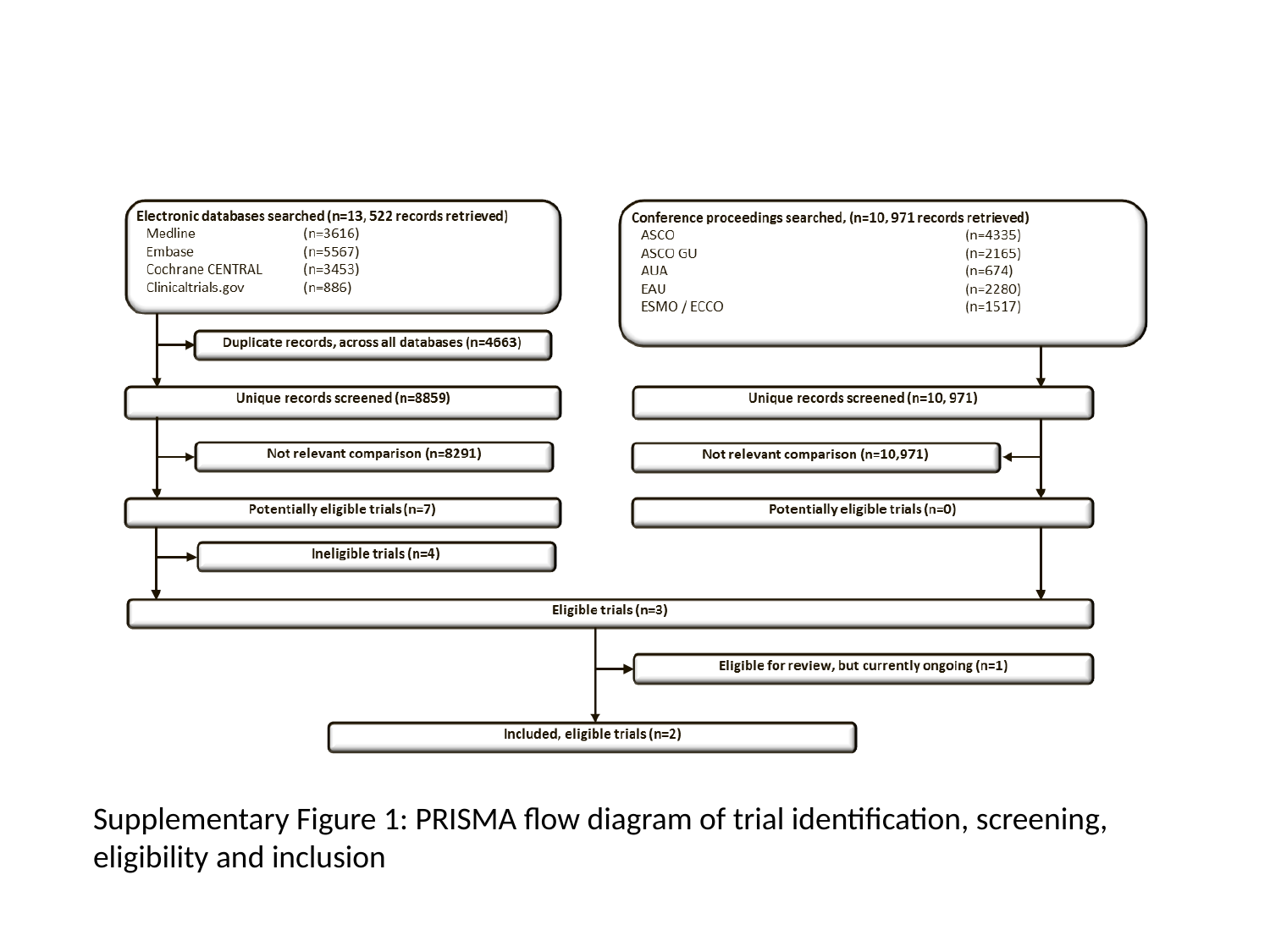

Supplementary Figure 1: PRISMA flow diagram of trial identification, screening, eligibility and inclusion
