# Supplementary material for: Prostate Radiotherapy for Metastatic Hormone-sensitive Prostate Cancer: A STOPCAP Systematic Review and Meta-analysis
Source: Eur Urol. 2019 Jul;76(1):115–24. doi: 10.1016/j.eururo.2019.02.003 (PMC6575150; doi:10.1016/j.eururo.2019.02.003)
Supplement: Supplementary file 4 [file mmc4.pptx]

## Slide 1
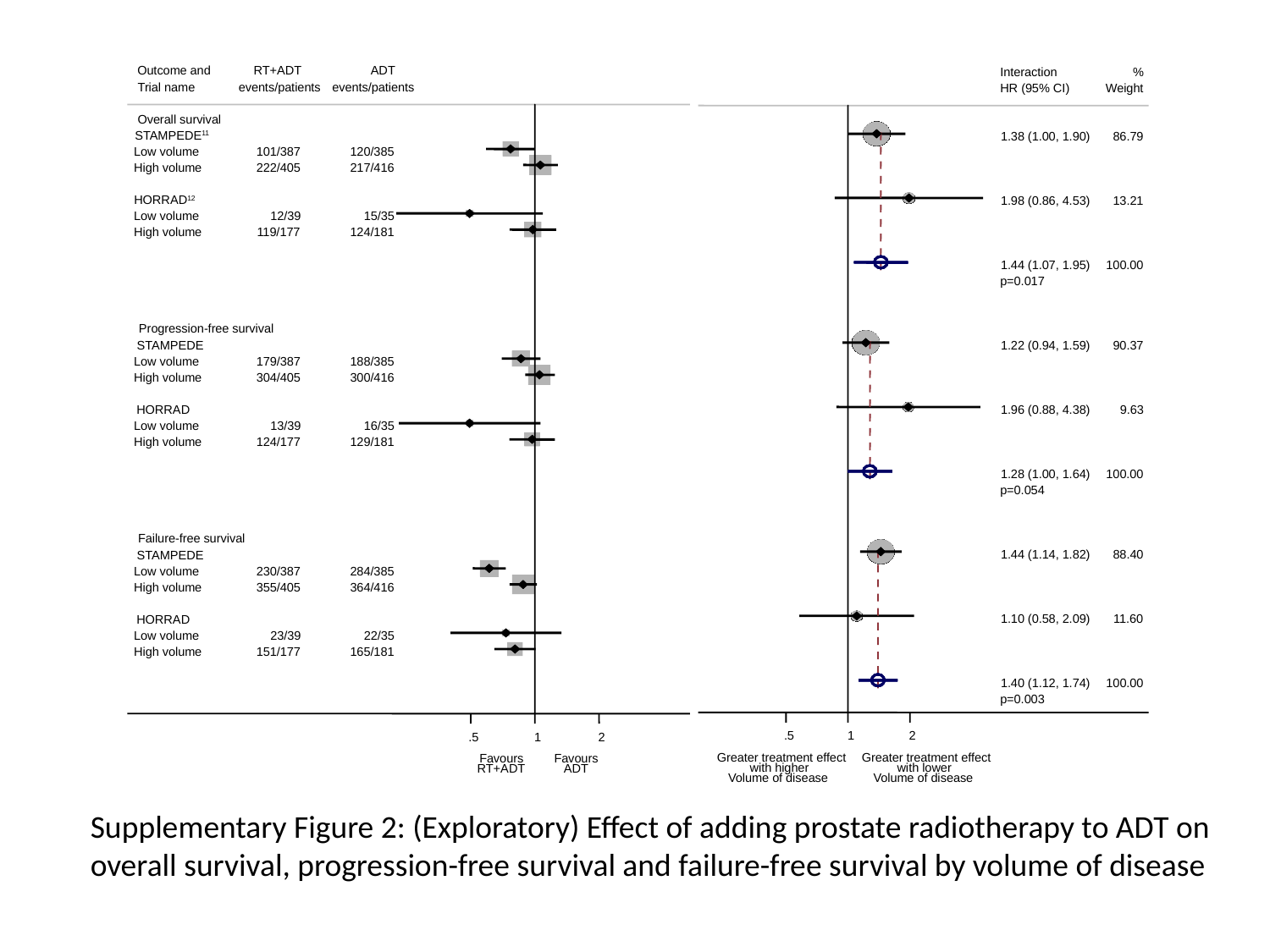

Outcome and
RT+ADT
ADT
Interaction
%
Trial name
events/patients
events/patients
HR (95% CI)
Weight
Overall survival
STAMPEDE11
1.38 (1.00, 1.90)
86.79
Low volume
101/387
120/385
High volume
222/405
217/416
HORRAD12
1.98 (0.86, 4.53)
13.21
Low volume
12/39
15/35
High volume
119/177
124/181
1.44 (1.07, 1.95)
100.00
p=0.017
Progression-free survival
STAMPEDE
1.22 (0.94, 1.59)
90.37
Low volume
179/387
188/385
High volume
304/405
300/416
1.96 (0.88, 4.38)
9.63
HORRAD
Low volume
13/39
16/35
High volume
124/177
129/181
1.28 (1.00, 1.64)
100.00
p=0.054
Failure-free survival
1.44 (1.14, 1.82)
88.40
STAMPEDE
Low volume
230/387
284/385
High volume
355/405
364/416
1.10 (0.58, 2.09)
11.60
HORRAD
Low volume
23/39
22/35
High volume
151/177
165/181
1.40 (1.12, 1.74)
100.00
p=0.003
.5
1
2
.5
1
2
Greater treatment effect
Greater treatment effect
Favours
Favours
with higher
with lower
RT+ADT
ADT
Volume of disease
Volume of disease
Supplementary Figure 2: (Exploratory) Effect of adding prostate radiotherapy to ADT on overall survival, progression-free survival and failure-free survival by volume of disease
